# Development and external validation of a simple pre-biopsy risk stratification model for clinically significant prostate cancer in men with PI-RADS 3 lesions: a multicenter transperineal biopsy study

**DOI:** 10.3389/fonc.2026.1864100

**Published:** 2026-09-04

**Authors:** Xuhui Peng, Xinyu Yi, Shan Tang

**Affiliations:** 1 Department of Urology, Shaoyang Central Hospital, Shaoyang, Hunan, China; 2 Department of Urology, Xiangtan Central Hospital (Hunan University Affiliated Hospital), Xiangtan, Hunan, China

**Keywords:** clinically significant prostate cancer, external validation, PI-RADS 3, prostate-specific antigen density, risk stratification

## Abstract

**Objective:**

PI-RADS 3 lesions are equivocal on prostate MRI, complicating biopsy decisions. We aimed to develop and externally validate a simple pre-biopsy risk stratification model using routinely available clinical and MRI variables to guide selective biopsy.

**Methods:**

In this multicenter retrospective study, men with PI-RADS 3 lesions undergoing transperineal biopsy at two tertiary hospitals (Jan 2023–Dec 2025) were included. One center served as the development cohort and the other as external validation. Clinically significant prostate cancer (csPCa) was defined as ISUP Grade Group ≥2. Candidate predictors included age, abnormal digital rectal examination (DRE), PSA density (PSAD), lesion location, and maximum lesion diameter. A multivariable logistic regression model was developed and validated externally.

**Results:**

Among 275 men (153 development, 122 validation), csPCa was detected in 37 (24.2%) and 31 (25.4%), respectively. PSAD and age were the main predictors, with other variables contributing to overall risk estimation. The model showed good discrimination (AUC 0.818 development, 0.846 validation), acceptable calibration, and decision curve analysis indicated potential clinical utility.

**Conclusion:**

This preliminary model using routinely available clinical and MRI variables may support pre-biopsy risk stratification in men with PI-RADS 3 lesions. However, further validation in larger and more diverse cohorts is required before routine clinical implementation.

## 1 Introduction

Prostate cancer remains one of the most commonly diagnosed malignancies in men worldwide and continues to impose a substantial health burden. According to the GLOBOCAN 2022 estimates, prostate cancer is the second most frequently diagnosed cancer among men globally, highlighting the continuing need for more accurate and efficient diagnostic pathways that can improve detection of clinically significant disease while minimizing overdiagnosis and overtreatment (1).

In recent years, multiparametric magnetic resonance imaging (mpMRI) has become central to the diagnostic work-up of men with suspected prostate cancer. The Prostate Imaging Reporting and Data System version 2.1 (PI-RADS v2.1) was developed to standardize prostate MRI acquisition, interpretation, and reporting, thereby improving communication between radiologists and urologists and facilitating risk stratification before biopsy (2). Current European Association of Urology (EAU) guidelines recommend performing MRI before prostate biopsy in men with suspected organ-confined disease, reflecting the growing role of MRI-directed diagnostic pathways in contemporary practice (3).

However, the management of PI-RADS 3 lesions remains a major clinical challenge. By definition, PI-RADS 3 represents an equivocal category, and this intermediate-risk group continues to create uncertainty in daily practice. A meta-analysis of PI-RADS v2.1 reported that the patient-level detection rate of clinically significant prostate cancer (csPCa) was approximately 20% for PI-RADS 3 lesions, which is substantially lower than that for PI-RADS 4–5 lesions but still high enough that blanket biopsy or blanket surveillance may both be inappropriate for some patients (4). The EAU guideline risk-adapted matrix further demonstrates marked heterogeneity within PI-RADS 3 lesions according to PSA density (PSAD), with csPCa risk ranging from very low to nearly 30% across PSAD strata, indicating that PI-RADS 3 lesions should not be managed as a uniform group (3).

Several studies have therefore explored whether routinely available clinical parameters can refine biopsy decisions in men with equivocal MRI findings. In a large multi-institutional collaborative study, Drevik et al. found that men with solitary PI-RADS 3 lesions had an overall csPCa rate of 20.0%, and higher PSAD was independently associated with csPCa; however, even with a PSAD cutoff of 0.15 ng/mL/cc, some clinically significant cancers would still be missed (5). Similarly, Schoots and Padhani proposed a risk-adapted biopsy strategy integrating MRI findings and PSAD, arguing that biopsy decisions after MRI should be individualized rather than based on MRI category alone (6). More recently, lesion location has also emerged as a potentially relevant factor, with evidence suggesting that isolated peripheral-zone PI-RADS 3 lesions may carry higher csPCa risk than transition-zone lesions, although current data remain limited and external validation is sparse (7).

Taken together, the existing literature suggests that PI-RADS 3 lesions represent a clinically important “gray zone” in which a simple, practical, and externally validated prediction model may help reduce unnecessary biopsies without substantially compromising csPCa detection. Nevertheless, many published studies are either single-center, based on limited variables, focused on a single marker such as PSAD, or lack robust external validation. In addition, models that are too complex may be difficult to implement in routine urological practice, especially in real-world multicenter settings.

Therefore, the present study aimed to develop and externally validate a pragmatic pre-biopsy risk stratification model for predicting csPCa in men with PI-RADS 3 lesions using routinely available clinical and MRI-derived variables. Rather than positioning the model as a tool to categorically avoid biopsy, we sought to evaluate whether it could support more selective biopsy decision-making by identifying men at relatively lower risk of csPCa. We hypothesized that a simple model derived in one center and tested in an independent external cohort would provide clinically useful risk stratification in this diagnostically challenging subgroup.

## 2 Materials and methods

### 2.1 Study design and patient selection

This was a multicenter retrospective study conducted at two tertiary hospitals between January 2023 and December 2025. Consecutive men with suspected prostate cancer who underwent pre-biopsy prostate magnetic resonance imaging (MRI) followed by transperineal prostate biopsy were screened for eligibility. The inclusion criteria were as follows: 1) presence of a PI-RADS 3 lesion on pre-biopsy MRI; 2) receipt of transperineal prostate biopsy at one of the participating centers;3) availability of complete pre-biopsy clinical, imaging, and pathological data; 4) age ≥18 years. The exclusion criteria were as follows: 1) prior diagnosis of prostate cancer; 2) previous treatment for prostate cancer, including radical prostatectomy, radiotherapy, androgen deprivation therapy, or focal therapy; 3) prior prostate surgery or other interventions that could substantially affect MRI interpretation or biopsy pathology; 4) incomplete data for key study variables; 5) non-diagnostic pathological results; 6) absence of definitive biopsy pathology for outcome classification.

Patients from Shaoyang Central Hospital were assigned to the development cohort, whereas patients from Xiangtan Central Hospital served as the external validation cohort. The aim of the study was to develop and externally validate a simple and clinically applicable pre-biopsy risk stratification model for predicting clinically significant prostate cancer (csPCa) in men with PI-RADS 3 lesions.

### 2.2 MRI acquisition and image assessment

All patients underwent pre-biopsy prostate MRI according to institutional protocols. MRI findings were interpreted by experienced genitourinary radiologists at each participating center, and lesions were scored using PI-RADS version 2.1 (2). Only patients with a PI-RADS 3 lesion were included in the present study.

MRI-derived variables were collected from the index lesion. In patients with multiple lesions, the lesion considered most relevant for biopsy decision-making according to the original MRI report was selected as the index lesion. The following imaging variables were extracted: lesion location and maximum lesion diameter. Lesion location was categorized as peripheral zone or non-peripheral zone. Maximum lesion diameter was recorded in millimeters.

Prostate volume was obtained from MRI-based measurements according to routine institutional practice and was used for the calculation of PSA density.

### 2.3 Biopsy procedure and pathological evaluation

The manuscript states that MRI and transperineal biopsy were performed according to institutional practice, but important technical details are missing. Please specify MRI magnet strength (1.5T or 3T), whether endorectal coils were used, MRI-targeted biopsy technique (cognitive, software fusion, or in-bore), the number of targeted and systematic cores obtained, and whether all patients underwent combined targeted plus systematic biopsy. These details are essential for reproducibility and interpretation of the diagnostic performance.

The primary study outcome was clinically significant prostate cancer (csPCa), defined as ISUP Grade Group ≥2. Patients with benign findings or ISUP Grade Group 1 disease were classified as non-csPCa.

### 2.4 Candidate predictors and variable definitions

The model was intentionally designed to remain simple and clinically practical. Based on clinical relevance, literature review, and routine availability before biopsy, five pre-specified candidate predictors were selected: age, abnormal digital rectal examination (DRE), prostate-specific antigen density (PSAD), lesion location, and maximum lesion diameter.

Age was analyzed as a continuous variable. DRE findings were categorized as normal or abnormal. PSAD was calculated as serum total prostate-specific antigen divided by prostate volume. Prostate volume was derived from MRI-based measurements according to routine practice at each center. Lesion size was defined as the maximum diameter of the index PI-RADS 3 lesion on MRI.

All five candidate predictors were pre-specified before model fitting based on clinical relevance, routine pre-biopsy availability, consistency of measurement across the two participating centers, and the intention to maintain model parsimony. No automated or stepwise variable-selection procedure was used. Family history of prostate cancer, quantitative apparent diffusion coefficient values, race or ethnicity, and additional serum or urinary biomarkers were not routinely recorded in the retrospective databases and therefore could not be evaluated. Prostate volume was incorporated into the calculation of PSAD and was not simultaneously entered as a separate predictor to avoid redundancy. Although previous biopsy status was recorded as a binary baseline variable, detailed information regarding the timing, sampling scheme, biopsy route, and targeting technique of previous biopsies was unavailable; therefore, it was not included among the five pre-specified predictors.

### 2.5 Model development and external validation

The prediction model was developed in the development cohort using multivariable logistic regression analysis. Baseline characteristics and candidate predictors were first compared between patients with and without csPCa within the development cohort. Given the pre-specified and clinically oriented design of the study, all five selected variables were entered into the multivariable logistic regression model. Odds ratios (ORs) and 95% confidence intervals (CIs) were calculated.

The final model was then fixed and applied to the external validation cohort without re-estimation of coefficients.

### 2.6 Model performance assessment

Model performance was evaluated in both the development and external validation cohorts. Discrimination was assessed using the area under the receiver operating characteristic curve (AUC). Calibration was evaluated using calibration plots to assess agreement between predicted and observed probabilities. Clinical utility was explored using decision curve analysis (DCA).Calibration was further quantified using calibration-in-the-large, calibration slope, and the Brier score. The 95% confidence intervals for model performance were estimated using 2,000 bootstrap resamples. At illustrative risk thresholds of 5% and 10%, we calculated the number and proportion of biopsies that could potentially be deferred, together with the corresponding number of missed csPCa cases.

To reflect the intended clinical application of the model, its potential value was interpreted as support for more selective biopsy decision-making rather than as an absolute rule for biopsy avoidance. In particular, the model was assessed for its ability to identify patients at relatively lower risk of csPCa who might be considered for more individualized decision-making.

### 2.7 Statistical analysis

Continuous variables were expressed as mean ± standard deviation or median with interquartile range, as appropriate, and categorical variables were presented as frequencies and percentages. Comparisons between groups were performed using the independent-samples t test or Mann–Whitney U test for continuous variables, depending on data distribution, and the chi-square test or Fisher’s exact test for categorical variables.

In the development cohort, univariable logistic regression analysis was first used to explore the association between each candidate predictor and csPCa. Multivariable logistic regression was then performed to construct the final model. Model performance was subsequently examined in both the development and external validation cohorts.

The linearity of continuous predictors on the logit scale was evaluated using restricted cubic spline terms and likelihood-ratio tests comparing nonlinear and linear specifications. Multicollinearity was assessed using variance inflation factors. Model convergence and potentially influential observations were evaluated using regression diagnostics, including Cook’s distance. Internal validation was performed using 2,000 bootstrap resamples to estimate optimism-corrected discrimination. The number of outcome events relative to the number of predictor parameters was also reported. A *post hoc* sample-size adequacy assessment was performed using the Riley/pmsampsize framework, assuming five predictor parameters, an outcome prevalence of 24.2%, and an anticipated C-statistic of 0.80. A uniform shrinkage factor was estimated as the mean calibration slope obtained from 2,000 bootstrap resamples. The non-intercept regression coefficients were multiplied by this factor, and the intercept was re-estimated to preserve the overall outcome prevalence.

All statistical analyses were performed using SPSS version 26.0 (IBM Corp., Armonk, NY, USA) and R software version 4.3.2 (R Foundation for Statistical Computing, Vienna, Austria). A two-sided *P* value <0.05 was considered statistically significant.

## 3 Results

**Figure 1:**
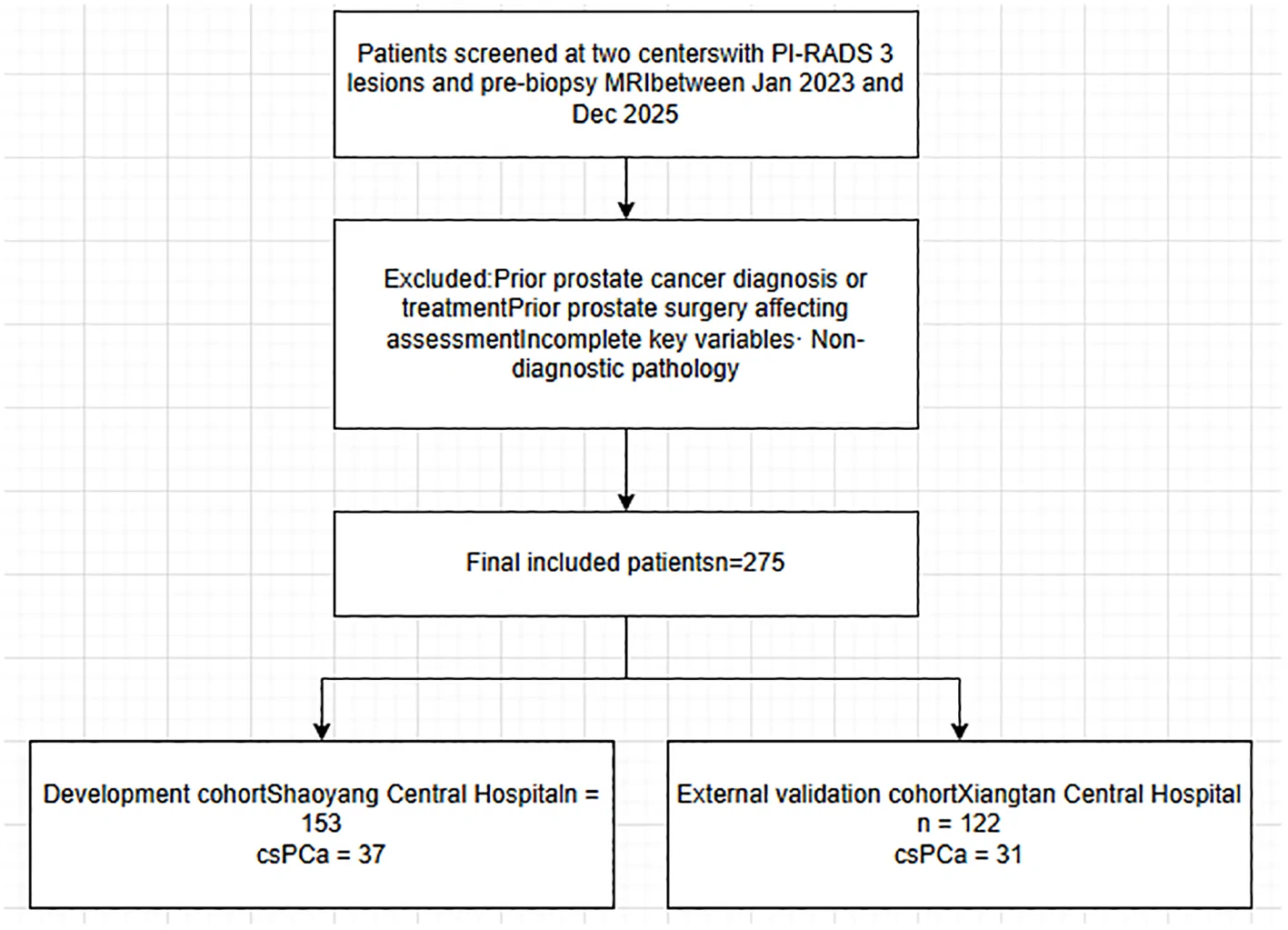
Flowchart of patient selection.

A total of 275 men with PI-RADS 3 lesions were included in the study. The patient selection process is shown in **Figure 1**. Of these, 153 patients from Shaoyang Central Hospital were assigned to the development cohort, and 122 patients from Xiangtan Central Hospital constituted the external validation cohort. Clinically significant prostate cancer (csPCa) was identified in 37 patients (24.2%) in the development cohort and 31 patients (25.4%) in the external validation cohort.

The baseline characteristics of the two cohorts are summarized in **Table 1**. Overall, the development and validation cohorts were broadly comparable with respect to age, serum PSA parameters, lesion size, PSA density, prior biopsy history, DRE findings, lesion location, and csPCa detection rate (all *P*>0.05). A significant difference was observed only for prostate volume, which was lower in the external validation cohort than in the development cohort (73.85 ± 23.24 mL vs. 79.95 ± 23.33 mL, *P* = 0.032).

**Table 1:** Baseline characteristics of the development and external validation cohorts.

Variable	Development cohort (n=153)	External validation cohort (n=122)	*P* value
Age, years	64.00 ± 5.04	64.61 ± 4.45	0.291
tPSA, ng/mL	11.92 ± 5.81	11.12 ± 5.56	0.244
fPSA, ng/mL	2.05 ± 1.26	1.89 ± 1.15	0.275
Prostate volume, mL	79.95 ± 23.33	73.85 ± 23.24	0.032
Lesion size, mm	11.71 ± 3.64	11.39 ± 3.90	0.491
PSAD, ng/mL/mL	0.148 ± 0.054	0.149 ± 0.053	0.894
Prior biopsy, n (%)	42 (27.5)	25 (20.5)	0.232
Abnormal DRE, n (%)	38 (24.8)	28 (23.0)	0.825
Peripheral-zone lesion, n (%)	87 (56.9)	65 (53.3)	0.637
csPCa, n (%)	37 (24.2)	31 (25.4)	0.925

Within the development cohort, univariable logistic regression analysis showed that PSA density (PSAD) was the strongest predictor of csPCa. Age showed a borderline positive association with csPCa risk, whereas abnormal digital rectal examination (DRE), lesion location, and lesion size were not significantly associated with csPCa.

A multivariable logistic regression model was then constructed using the five pre-specified variables: age, abnormal DRE, PSAD, lesion location, and maximum lesion diameter. In the adjusted model, PSAD remained the dominant independent predictor of csPCa (OR 12.43, 95% CI 4.60–33.57, *P* < 0.001, per 0.1 ng/mL/mL increase), whereas the effects of age, DRE status, lesion location, and lesion size were attenuated after adjustment. Although these variables were not independently significant in the final model, they were retained because of their clinical relevance and contribution to overall risk estimation. The detailed regression results are shown in **Table 2**.

**Table 2:** Univariable and multivariable logistic regression analyses for predicting clinically significant prostate cancer in the development cohort.

Variable	(95% CI)	*P* value	Multivariable OR (95% CI)	*P* value
Age, years	1.078 (0.999–1.163)	0.053	1.067 (0.978–1.163)	0.144
Abnormal DRE	1.662 (0.735–3.754)	0.222	1.264 (0.473–3.378)	0.640
PSAD (per 0.1 ng/mL/mL increase)	12.653 (4.929–32.481)	<0.001	12.427 (4.600–33.572)	<0.001
Peripheral-zone lesion	1.335 (0.625–2.850)	0.456	1.360 (0.550–3.361)	0.506
Lesion size, mm	1.068 (0.964–1.183)	0.210	0.979 (0.867–1.106)	0.730

Thank you for this important comment. We have added the complete logistic regression equation, including the intercept, regression coefficients, variable coding, and probability calculation, to the Results section, allowing readers to calculate individual predicted probabilities.

There was no significant evidence of nonlinearity for age (*P* = 0.127), PSAD (*P* = 0.166), or lesion size (*P* = 0.940). VIFs ranged from 1.01 to 1.10, indicating no meaningful multicollinearity. The development cohort contained 37 csPCa events for five predictor parameters, corresponding to an events-per-parameter ratio of 7.4. A *post hoc* sample-size assessment indicated that approximately 282 participants, including 68 csPCa events, were estimated to be required. Therefore, the available development cohort of 153 patients and 37 events was smaller than recommended. The bootstrap-derived uniform shrinkage factor was 0.870 and was applied to the regression coefficients to reduce potential overfitting.

After bootstrap-based coefficient shrinkage and intercept re-estimation, the final prediction equation was: logit(P) = −8.3048 + 0.0562 × age + 0.2039 × abnormal DRE + 2.1920 × (PSAD/0.1) + 0.2674 × peripheral-zone lesion − 0.0186 × lesion size. Abnormal DRE and peripheral-zone lesion were coded as 1, whereas normal DRE and non-peripheral-zone lesion were coded as 0. The predicted probability of csPCa was calculated as P = exp[logit(P)]/{1 + exp[logit(P)]}.

**Figure 2:**
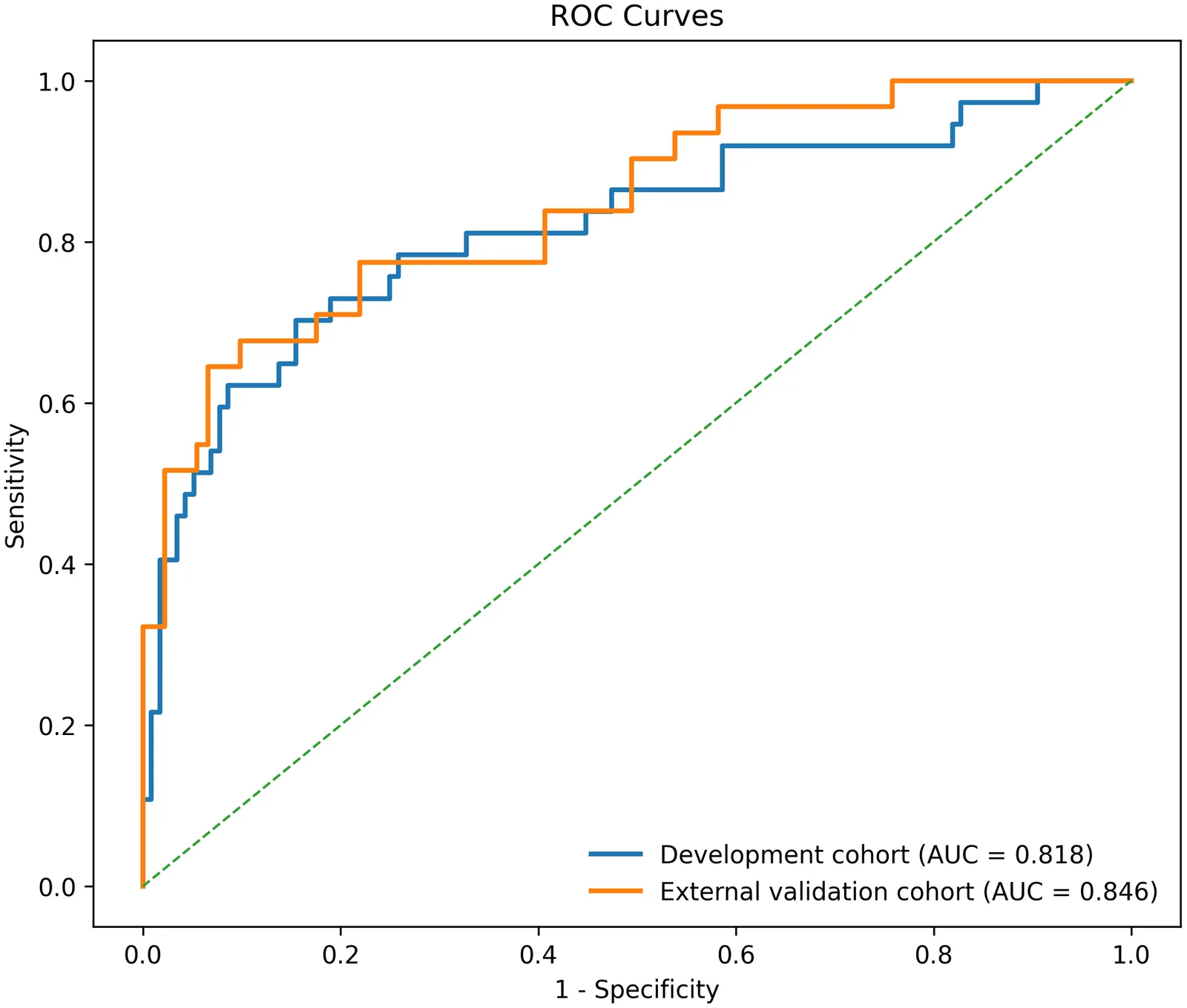
Receiver operating characteristic curves of the model in the development and external validation cohorts.

The receiver operating characteristic curves of the model in the development and external validation cohorts are shown in **Figure 2**. In the development cohort, the model demonstrated good discrimination for predicting csPCa, with an area under the receiver operating characteristic curve (AUC) of 0.818. After 2,000 bootstrap resamples, the estimated optimism in the AUC was 0.027, resulting in an optimism-corrected AUC of 0.790. When applied to the independent external validation cohort, the model retained favorable discriminatory performance, with an AUC of 0.846. These findings suggest that the model retained predictive performance across the two participating centers and may be transportable to similar clinical settings.

**Figure 3:**
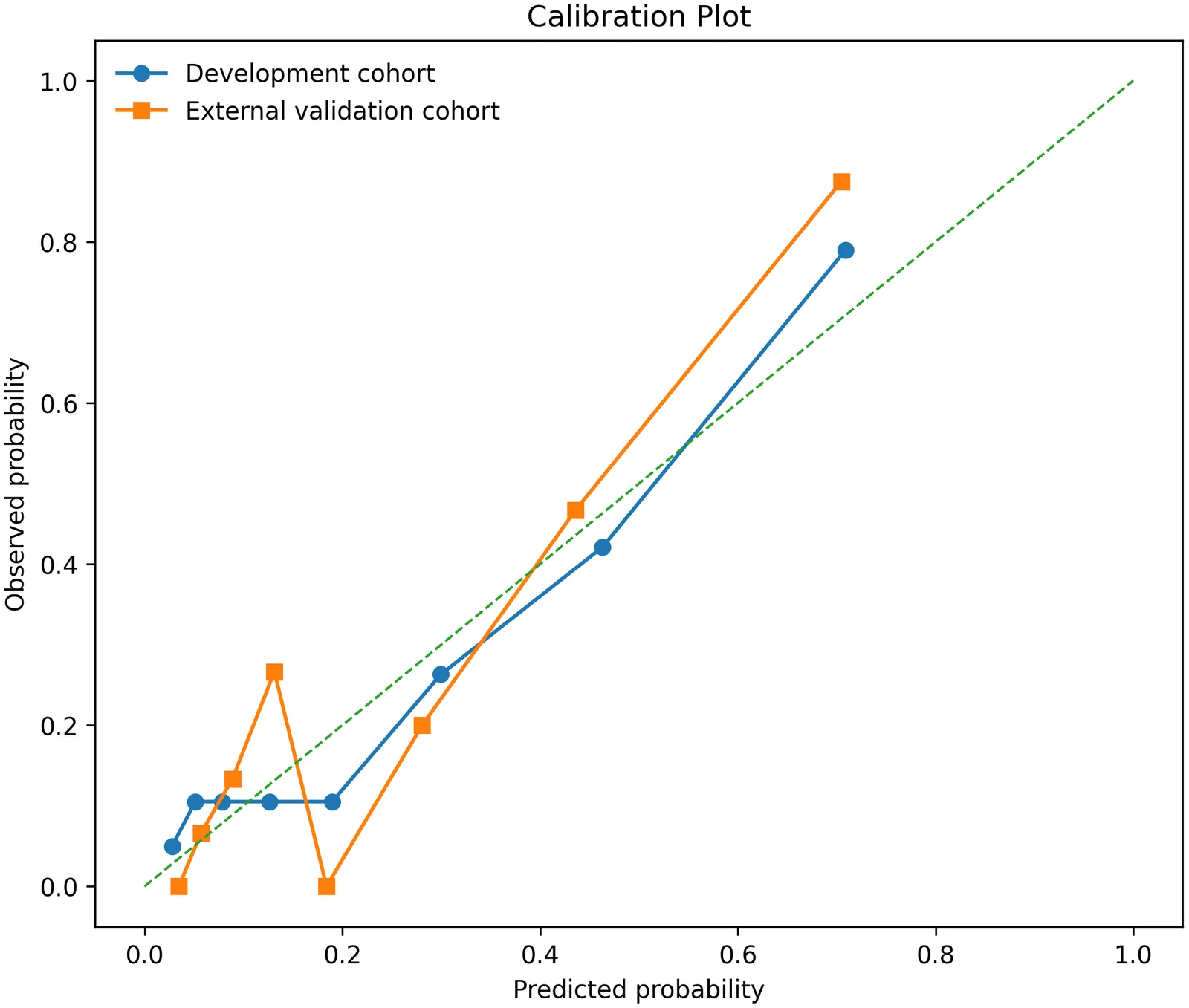
Calibration plots of the model in the development and external validation cohorts.

Calibration plots for the development and validation cohorts are shown in **Figure 3**. The model demonstrated acceptable agreement between predicted and observed probabilities in both cohorts, suggesting that the estimated risks were reasonably consistent with actual biopsy outcomes.

**Figure 4:**
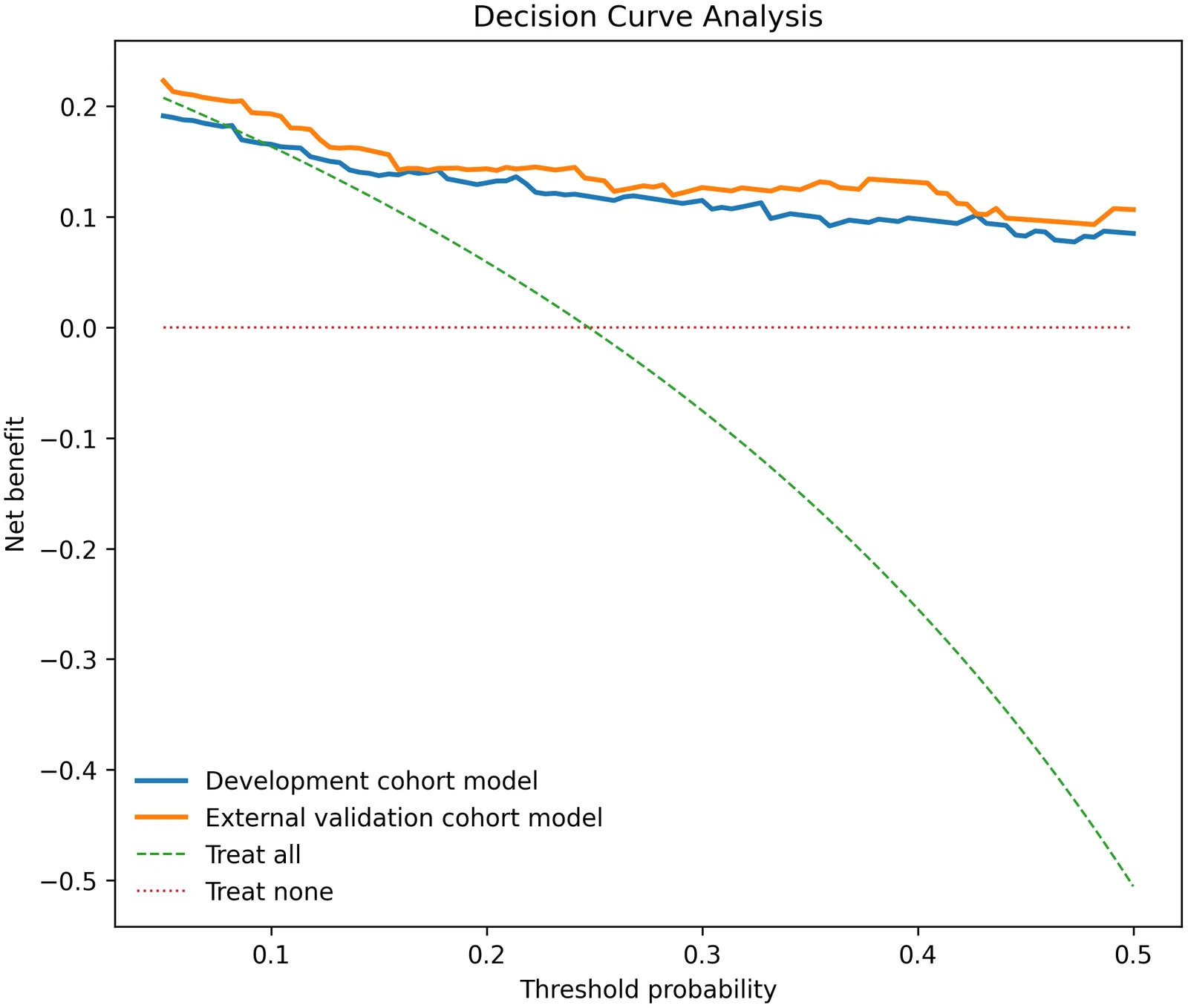
Decision curve analysis of the model in the development and external validation cohorts.

Decision curve analysis is presented in **Figure 4**. Across a range of clinically relevant threshold probabilities, the model provided a net benefit over default strategies, supporting its potential clinical usefulness in men with PI-RADS 3 lesions. At a 5% predicted-risk threshold, biopsy could potentially have been deferred in 29 of 153 patients (19.0%) in the development cohort and 19 of 122 patients (15.6%) in the validation cohort, with 3 of 37 (8.1%) and 0 of 31 (0%) csPCa cases missed, respectively. At a 10% threshold, the corresponding biopsy-reduction rates were 39.9% (61/153) and 36.1% (44/122), with 5 of 37 (13.5%) and 2 of 31 (6.5%) csPCa cases missed, respectively. These thresholds are presented as illustrative trade-offs rather than definitive criteria for biopsy omission.

## 4 Discussion

In this multicenter retrospective study, we developed and externally validated a simple pre-biopsy risk stratification model for clinically significant prostate cancer (csPCa) in men with PI-RADS 3 lesions. The main finding was that a model based on routinely available clinical and MRI-derived variables showed acceptable discrimination and retained predictive performance in an independent external validation cohort. These results suggest that simple risk stratification may provide clinically useful support for more selective biopsy decision-making in this diagnostically challenging subgroup.

The management of PI-RADS 3 lesions remains problematic in daily practice because this category represents an equivocal MRI finding with substantial heterogeneity in csPCa risk. Previous studies have emphasized that PI-RADS 3 lesions remain one of the major unresolved areas in MRI-based prostate cancer diagnostics and that biopsy decisions should not rely on MRI category alone but instead be refined using additional clinical parameters (8). This was the main clinical rationale for the present study. Rather than developing a complex prediction tool, we focused on a small number of routinely available variables that are more likely to be applicable in real-world settings.

Among the candidate predictors, PSA density emerged as the most important variable in our model. This finding is consistent with previous studies showing that PSA density provides meaningful diagnostic refinement in men with equivocal MRI lesions (9, 10). More technically demanding approaches, such as radiomics, have also been investigated in PI-RADS 3 lesions and may further improve risk stratification (11–13). However, these methods are not yet as accessible or easily applicable as models based on standard pre-biopsy variables. For this reason, we deliberately chose a pragmatic and clinically interpretable model structure.

An important strength of the present study is the use of external validation across two centers. Many prediction studies in this field remain limited to single-center analyses or internal validation only, which reduces confidence in generalizability. External validation has increasingly been recognized as a key requirement for clinically credible prediction models in men with PI-RADS 3 lesions (14, 15). From a practical perspective, our model should not be regarded as a tool to categorically omit biopsy, but rather as a decision-support instrument that may help identify men at relatively lower risk of csPCa and support more individualized biopsy discussions.

Several PI-RADS 3-specific prediction models have previously been reported. Hu et al. developed an externally validated nomogram in biopsy-naïve men with PSA <10 ng/mL using clinical and MRI-derived variables, while Liang et al. developed zone-specific nomograms in a larger biparametric MRI cohort (14, 15). Osses et al. evaluated a relatively simple risk-stratification approach incorporating routinely available clinical and MRI variables, while Mjaess et al. further refined clinically relevant PSAD thresholds for biopsy decision-making (16, 17). Compared with these studies, our model does not introduce entirely novel predictors. Its contribution lies primarily in its pragmatic five-variable structure, inclusion of patients without a predefined PSA restriction, exclusive use of the transperineal biopsy route, and independent center-level validation. Therefore, the present findings should be interpreted as a pragmatic and confirmatory contribution to PI-RADS 3 risk stratification.

Recent evidence has also suggested that lesion location may be relevant in PI-RADS 3 lesions, particularly with respect to peripheral- versus transition-zone disease (18). In our study, lesion location did not emerge as a dominant adjusted predictor, but it was retained because it is clinically intuitive and may still contribute to overall risk estimation. This reflects the pragmatic intent of our model: the goal was not to identify a single dominant etiological factor, but to provide a clinically usable tool for integrated pre-biopsy risk assessment.

Several limitations should be acknowledged. First, this was a retrospective study and was therefore susceptible to selection bias and information bias. In addition, the requirement for complete clinical, imaging, and pathological data may have introduced a complete-case selection effect. Second, despite the multicenter design, the overall sample size remained modest. The development cohort contained only 37 csPCa events for five predictor parameters, corresponding to an events-per-parameter ratio of 7.4. Although bootstrap internal validation showed that the model retained acceptable discrimination after optimism correction, the limited number of events may have affected coefficient stability and increased the risk of overfitting. Therefore, the model should be regarded as preliminary and requires further validation in larger populations. Third, several potentially informative predictors, including family history, race or ethnicity, quantitative ADC measurements, and additional serum or urinary biomarkers, were not routinely recorded and could not be evaluated. Although previous biopsy status was available as a binary variable, detailed characteristics of prior biopsy procedures were unavailable. Prostate volume was represented through PSAD rather than modeled separately. These decisions preserved model simplicity and cross-center consistency but may have excluded predictors with incremental value. Fourth, MRI examinations were interpreted independently at each center without centralized image review or formal assessment of inter-reader agreement. Therefore, PI-RADS classification, lesion location, and lesion diameter may have been affected by inter-reader variability. Although both centers used 1.5-T MRI without an endorectal coil and a standardized 12-core cognitive MRI-directed transperineal biopsy protocol, differences among radiologists, biopsy operators, and local implementation may still have introduced center-related heterogeneity. Fifth, all analyzed patients underwent biopsy and had definitive pathological outcomes, which reduced partial verification bias within the study cohort. Nevertheless, selection or verification-related bias cannot be completely excluded because only men who proceeded to biopsy were included. Finally, both participating institutions were tertiary hospitals located in the same geographic region, which may limit the generalizability of the findings to other populations, community settings, and healthcare systems. The model should therefore be regarded as a decision-support tool rather than a universal rule for biopsy omission.

In conclusion, we developed and externally validated a simple pre-biopsy risk stratification model for csPCa in men with PI-RADS 3 lesions. Using routinely available variables, the model may serve as a pragmatic aid for more selective biopsy decision-making. Further prospective multicenter studies are needed to confirm its calibration, optimize clinically relevant thresholds, and determine its impact on real-world biopsy practice.

## Data Availability

The raw data supporting the conclusions of this article will be made available by the authors, without undue reservation.

## References

[1] Bray F Laversanne M Sung H Ferlay J Siegel RL Soerjomataram I . Global cancer statistics 2022: GLOBOCAN estimates of incidence and mortality worldwide for 36 cancers in 185 countries. CA Cancer J Clin. (2024) 74:229–63. doi: 10.3322/caac.21834 38572751

[2] Turkbey B Rosenkrantz AB Haider MA Padhani AR Villeirs G Macura KJ . Prostate imaging reporting and data system version 2.1: 2019 update of prostate imaging reporting and data system version 2. Eur Urol. (2019) 76:340–51. doi: 10.1016/j.eururo.2019.02.033 30898406

[3] EAU Guidelines . Edn. Presented at the EAU Annual Congress London 2026. Arnhem, The Netherlands: EAU Guidelines Office (2026).

[4] Oerther B Engel H Bamberg F Sigle A Gratzke C Benndorf M . Cancer detection rates of the PI-RADSv2.1 assessment categories: systematic review and meta-analysis on lesion level and patient level. Prostate Cancer Prostatic Dis. (2022) 25:256–63. doi: 10.1038/s41391-021-00417-1 34230616 PMC9184264

[5] Drevik J Dalimov Z Guzzo T Belkoff L Marlowe B Raman J . Utility of PSA density in patients with PI-RADS 3 lesions across a large multi-institutional collaborative. Urol Oncol. (2022) 40:490.e1–6. doi: 10.1016/j.urolonc.2022.08.009 36163229

[6] Schoots IG Padhani AR . Risk-adapted biopsy decision based on prostate magnetic resonance imaging and prostate-specific antigen density for enhanced biopsy avoidance in first prostate cancer diagnostic evaluation. BJU Int. (2021) 127:175–8. doi: 10.1111/bju.15277 33089586 PMC7894174

[7] Schoots IG . MRI in early prostate cancer detection: how to manage indeterminate or equivocal PI-RADS 3 lesions? Transl Androl Urol. (2018) 7:70–82. doi: 10.21037/tau.2017.12.31 29594022 PMC5861283

[8] Nicola R Hectors SJ Vargas HA . PI-RADS 3 lesions: a critical review and discussion of how to improve management. Abdom Radiol (NY). (2023) 48:2272–87. doi: 10.1007/s00261-023-03929-7 37160472

[9] Wang S Kozarek J Russell R Drescher M Khan A Kundra V . Diagnostic performance of prostate-specific antigen density for detecting clinically significant prostate cancer in the era of magnetic resonance imaging: a systematic review and meta-analysis. Eur Urol Oncol. (2024) 7:189–203. doi: 10.1016/j.euo.2023.08.002 37640584 PMC12826839

[10] Rajendran I Lee KL Thavaraja L Barrett T . Risk stratification of prostate cancer with MRI and prostate-specific antigen density-based tool for personalized decision making. Br J Radiol. (2024) 97:113–9. doi: 10.1093/bjr/tqad027 38263825 PMC11027333

[11] Corsi A De Bernardi E Bonaffini PA Franco PN Nicoletta D Simonini R . Radiomics in PI-RADS 3 multiparametric MRI for prostate cancer identification: literature models re-implementation and proposal of a clinical-radiological model. J Clin Med. (2022) 11:6304. doi: 10.3390/jcm11216304 36362530 PMC9656103

[12] Li T Sun L Li Q Luo X Luo M Xie H . Development and validation of a radiomics nomogram for predicting clinically significant prostate cancer in PI-RADS 3 lesions. Front Oncol. (2022) 11:825429. doi: 10.3389/fonc.2021.825429 35155214 PMC8825569

[13] Zhao YY Xiong ML Liu YF Duan LJ Chen JL Xing Z . Magnetic resonance imaging radiomics-based prediction of clinically significant prostate cancer in equivocal PI-RADS 3 lesions in the transitional zone. Front Oncol. (2023) 13:1247682. doi: 10.3389/fonc.2023.1247682 38074651 PMC10701731

[14] Hu C Sun J Xu Z Zhang Z Zhou Q Xu J . Development and external validation of a novel nomogram to predict prostate cancer in biopsy-naïve patients with PSA <10 ng/ml and PI-RADS v2.1 = 3 lesions. Cancer Med. (2023) 12:2560–71. doi: 10.1002/cam4.5100 35920264 PMC9939143

[15] Liang Z Feng T Zhou Y Yang Y Sun Y Zhou Z . Nomograms for predicting clinically significant prostate cancer in men with PI-RADS-3 biparametric magnetic resonance imaging. Am J Cancer Res. (2024) 14:73–85. doi: 10.62347/XBBI9870 38323293 PMC10839314

[16] Osses DF Arsov C Schimmöller L Schoots IG van Leenders GJLH Esposito I . Equivocal PI-RADS three lesions on prostate magnetic resonance imaging: risk stratification strategies to avoid MRI-targeted biopsies. J Pers Med. (2020) 10:270. doi: 10.3390/jpm10040270 33321791 PMC7768373

[17] Mjaess G Haddad L Jabbour T Baudewyns A Bourgeno HA Lefebvre Y . Refining clinically relevant cut-offs of prostate specific antigen density for risk stratification in patients with PI-RADS 3 lesions. Prostate Cancer Prostatic Dis. (2025) 28:173–9. doi: 10.1038/s41391-024-00872-6 39048664

[18] Malshy K Ochsner A Ortiz R Ahn B Glebocki R Liu M . Comparison of the incidence of clinically significant prostate cancer in patients with isolated peripheral versus transitional zone PI-RADS 3 lesions. Urologia. (2025) 92:51–8. doi: 10.1177/03915603241286064 39344465

